# Social status and modern‐type depression: A review

**DOI:** 10.1002/brb3.1464

**Published:** 2019-11-19

**Authors:** Takashi Komori, Manabu Makinodan, Toshifumi Kishimoto

**Affiliations:** ^1^ Department of Psychiatry Nara Medical University School of Medicine Kashihara Japan

**Keywords:** *hikikomori*, modern‐type depression, motivation, social dominance, social status

## Abstract

**Backgrounds:**

Social hierarchy is one of the most influential social structures employed by social species. While dominants in such hierarchies can preferentially access rich resources, subordinates are forced into lower social statuses and lifestyles with inferior resources. Previous studies have indicated that the social rank regulates social behaviors and emotion in a variety of species, whereby individual organisms live within the framework of their ranks. However, in human societies, people, particularly young men, who cannot accept their own social status may show social withdrawal behaviors such as *hikikomori* to avoid confronting their circumstances.

**Methods:**

This article reviews the neural mechanisms underlying social status identified in animal studies with rodents and primates, and assesses how social rank affects animal's social behaviors and emotion which may be relevant to modern type depression.

**Results:**

Several brain regions such as medial prefrontal cortex are implicated in the formation of animal's social status, which leads to the differences in vulnerability and resilience to social stress.

**Conclusion:**

On the basis of these findings, we propose that physical interventions such as voluntary exercise, diet, transcranial direct current stimulation, and psychotherapy, rather than psychotropic drugs, may be useful therapeutic approaches for modern type depression, which is a typical example of social status conflict and a phenotype of adjustment disorder to the traditional hierarchical social order.

## Graphical abstract

This article reviews the neural mechanisms underlying social hierarchy identified in animal studies using rodents and primates and assesses how social ranks affect the animals' social behaviors and emotions. On the basis of these findings, we propose that physical interventions such as voluntary exercise, diet, and tDCS, as well as psychotherapy, rather than psychotropic drugs, may be useful as therapeutic approaches for MTD.

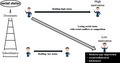

## 1 INTRODUCTION

Social hierarchies are essential for social species to maintain order in their societies by minimizing struggles over food, territory, and reproduction (Drews, 1993; Halevy, Y. Chou, & D. Galinsky, 2011; Sapolsky, 2005; Schjelderup‐Ebbe, 1922). The reduced competition owing to social hierarchy within the same species facilitates continuation of the species; however, social status can also have some negative influences within social species (Sapolsky, 2005). The battle for dominance results in the formation of dominants and subordinates among members of a species (Hand, 1986; Qu, Ligneul, Van der Henst, & Dreher, 2017), and these social statuses regulate the individual behaviors, performance, emotions, and health of the corresponding members (Koski, Xie, & Olson, 2015; Sapolsky, 2005; Tamashiro, Nguyen, & Sakai, 2005; Zhou, Sandi, & Hu, 2018). For instance, subordinates are forced to live with the social stress caused by repeated social defeat experiences in survival competitions, and this form of social stress induces various mental and physical health problems through disruption of the endocrine system, immune system, and brain functions (Cavigelli & Chaudhry, 2012; Chiao, 2010; Qu et al., 2017; Sapolsky, 2005; Tamashiro et al., 2005; Watanabe & Yamamoto, 2015; Zhou et al., 2018). In contrast, it is also known that, in some species, a high rank can also burden individuals. For instance, a high rank is costly and energetically demanding for male chimpanzees (Masataka et al., 1990). Moreover, dominance relationships frequently change between male crested macaques (Neumann et al., 2011). Thus, the effects of social ranks differ in species and populations (Sapolsky, 2005).

In human society, social status is essential for social communities such as families, schools, and workplaces (Halevy et al., 2011). Humans consciously or unconsciously accept their individual social ranks and act within the framework of their own social ranks (Halevy et al., 2011). However, people who cannot accept their own social rank tend to suffer from a sense of defeat (Rohde, 2001). Modern‐type depression (MTD) is a typical example of such social status conflicts and a phenotype of adjustment disorder, that is, maladaptation to the Japanese traditional hierarchical social order (Kato, Hashimoto, et al., 2016; Kato & Kanba, 2017). Patients with MTD show depression‐like behaviors that are similar to the phenotypes of subordinate mice, along with social status‐related characteristics such as learned helplessness, social avoidance, and anhedonia (Venzala, Garcia‐Garcia, Elizalde, Delagrange, & Tordera, 2012). Thus, research findings regarding social status may elucidate the mechanisms underlying MTD.

In this review, we present recent findings concerning the neural mechanisms underlying social status in various subjects, ranging from rodents to humans and discuss the potential relationships between social status and the pathobiology of MTD.

## 2 BIOLOGICAL ASPECTS IMPLICATED IN SOCIAL STATUS IN ANIMALS

### 2.1 Biological aspects implicated in social status in rodents

In rodents, behaviors that are regulated by social status have been used as a marker of social ranks (Wang, Kessels, & Hu, 2014; Zhou et al., 2018). The tube test is one behavioral experiment that is used to measure social rank (Lindzey, Winston, & Manosevitz, 1961; Wang et al., 2014; Zhou et al., 2018). Two rodents are allowed to enter a narrow tube from opposite sides and push the opponent to the other side. The rodent that beats the opponent is defined as the dominant. According to this test, dominants exhibit a stronger motivation to win than subordinates (Lindzey et al., 1961; Zhou et al., 2017). Recent studies have suggested that there are relationships between social dominance and various behaviors in laboratory rodents. Several studies have shown that dominant rodents display more aggressive/offensive behaviors than subordinates, while subordinate rodents display more submissive/defensive behaviors than dominants in their home cages (Blanchard & Blanchard, 1990; Blanchard et al., 1995; Wang et al., 2014, 2011). Moreover, some studies have indicated that dominant rodents display more reproductive behaviors than subordinates (Blanchard et al., 1995; Wang et al., 2011). In mice, the 70‐kHz ultrasonic vocalization emitted by male mice is a courtship (reproductive) behavior and has been assumed to reflect sexual motivation (Nyby, Dizinno, & Whitney, 1976; Whitney, Alpern, Dizinno, & Horowitz, 1974). Dominant mice emit more 70‐kHz ultrasonic vocalizations than subordinate mice, which indicates that dominant mice display greater motivation to reproduce (Blanchard et al., 1995; Wang et al., 2011). In addition to the findings on sexual motivation, some studies have also revealed the presence of relationships between social status and social motivation. For example, Kunkel and Wang (2018) administered the three‐chamber social interaction test, which is a behavioral experimental paradigm to measure sociality to dominant and subordinate mice. The authors found that dominant mice showed a greater motivation to approach novel mice than did subordinates, which could be indicative of social withdrawal in subordinate mice.

Social status affects emotions such as anxiety and depression‐like symptoms. Horii et al. (2017) measured anxiety levels in dominant and subordinate mice using the elevated plus‐maze test. The authors found that dominant mice spend more time in the open arms than subordinate mice, which indicates that the subordinate mice exhibited more anxiety than dominant mice. In contrast, using the elevated plus‐maze test and the open field test, Larrieu et al. (2017) revealed that dominant mice show more anxiety than subordinate mice. Interestingly, the authors found that anxiety levels were linearly correlated with social ranks in home cages. Larrieu et al. proposed that their findings were consistent with those of previous studies in which aggression (a property of dominance) and anxiety‐related behaviors were positively correlated (Larrieu et al., 2017). Although it remains unclear why the relationships between anxiety and dominance differ between these two studies, social status has been shown to affect anxiety in laboratory mice. Intriguingly, Horii et al. (2017) found that subordinate mice showed a longer duration of immobility (learned helplessness) in the forced swim test. This finding suggested that subordinate mice show depression‐like symptoms, including learned helplessness. Chronic social defeat stress (CSDS), a behavioral experimental paradigm in which a mouse is repeatedly attacked by a larger aggressive mouse, is an effective experimental method to force mice to become subordinates (Golden, Covington, Berton, & Russo, 2011). While the social battle to establish social status in home cages among mice from the same strain may be different from CSDS, in which a mouse is attacked by a larger mouse, subordinate mice show similar phenotypes as CSDS mice based on the shared context of social defeat. Namely, CSDS mice show depression‐like symptoms, including anhedonia, social withdrawal, and learned helplessness (Venzala et al., 2012), much like subordinate mice of the same strain in home cages (Horii et al., 2017).

### 2.2 Biological aspects implicated in social status in nonhuman primates

In primate societies, the history of winning or losing (success or defeat) in social competitions is a significant regulating factor of social status (Hsu, Earley, & Wolf, 2006; Qu et al., 2017; Zhou et al., 2018). Winners can preferentially access rich resources, such as food, sexual partners, and territory, which helps them to stay healthy (Sapolsky, 2005). Therefore, winners find it easier to win the next competition, while losers will continue to lose (Hsu et al., 2006). This phenomenon is called the “winner/loser effect,” which has a strong influence on the regulation of social status in many species (Hsu & Wolf, 1999; Trannoy, Penn, Lucey, Popovic, & Kravitz, 2016; Zilioli & Watson, 2014). Since survival resources (e.g., food, reproductive females) are limited in nature, social hierarchy enables the appropriate distribution of these resources and the continued existence of the species (Halevy et al., 2011). The hierarchy determines the priority within groups and forces individual organisms to live within the framework of their ranks (Hand, 1986). As mentioned above, this phenomenon is derived from the “winner/loser effect,” whereby dominants can preferentially access food, reproductive females, and territory, thereby allowing the species to leave superior offspring. Although social hierarchy is essential for the survival of social groups, it impairs physical and mental health, especially in subordinates (Sapolsky, 2005). Subordinates are forced to accept their social ranks and live their life under chronic social stress, which can threaten their health (Sapolsky, 2005; Tamashiro et al., 2005).

On the other hand, it has been reported that the experimental data of captive primates before they are placed in social groups do not predict their social rank in a social group (Morgan et al., 2000), and, in feral populations, social rank‐related stress depends on the social style and social organization of a species. For instance, high‐ranking individuals tend to experience stress in despotic hierarchies that are maintained through frequent physical reassertion of dominance (e.g., feral populations of dwarf mongooses, African wild dogs, female ring‐tailed lemurs), or in a society with an unstable social hierarchy (e.g., feral baboons) (Sapolsky, 2005). These findings indicate that social hierarchy in nonhuman primates is not formed as simply as rodents described above.

### 2.3 Biological aspects implicated in social status in humans

In humans, social hierarchy, especially in modern society, is evaluated using socioeconomic status (SES) in social psychological literatures (Cavigelli & Chaudhry, 2012; Manstead, 2018; Sapolsky, 2005) because it has a reasonable sense of where people belong, relative to others, in terms of economic factors and educational attainment, and in addition, traditional boundaries between social classes have become less manifest in modern society (Manstead, 2018). Farah (2017) proposed that there could be relationships between SES, brain structure and functions, and life outcomes. Indeed, SES has been implicated in human physical and mental health as well as human cognitive abilities (Farah, 2017, 2018; Sapolsky, 2005). A higher SES reduces the rates of heart disease, stroke, cancer, diabetes, and many other serious illnesses; thus, SES is positively related to longevity (Adler & Stewart, 2010). Similarly, a higher SES also reduces the rates of mental health problems such as depression, anxiety, and psychosis (McLaughlin, Costello, Leblanc, Sampson, & Kessler, 2012). SES largely affects individual health, and a higher SES has been associated with good physical and mental health. A higher SES is also positively associated with cognitive functions, for example, memory (Noble et al., 2015), working memory (Evans & Schamberg, 2009), and intelligence quotient (IQ) (von Stumm & Plomin, 2015). Thus, a higher SES and higher social rank are associated with good performance in various aspects in human society (Hackman & Farah, 2009; Sirin, 2005). In rodent studies, social dominance is determined by direct social interactions such as social competition (Wang et al., 2014). In contrast, in humans, the dominance and social rank are determined by relative social cues such as SES (Farah, 2017), and the “dominant trait” in humans is usually measured by computer‐based tasks in which people decide their relative dominance positions compared with a computerized player or another human player (Zink et al., 2008). A recent study elucidated some characteristics of dominant men (da Cruz et al., 2018), and these characteristics may be shaped by the “dominant personality trait,” which is defined as the presence of a motive to control others (Watanabe & Yamamoto, 2015).

## 3 NEURAL CIRCUITS UNDERLYING SOCIAL STATUS IN ANIMALS AND HUMANS

The formation of social hierarchy arises from repeated social activities between individuals, which is affected by environmental pressures (Chen & Hong, 2018). This consequently forms neural circuits that supposedly regulate behaviors and physiological responses of individuals (Miller et al., 2017; Munuera, Rigotti, & Salzman, 2018; Sapolsky, 2005). However, it remains unknown whether social status is represented by distinct neural substrates (Munuera et al., 2018). The medial prefrontal cortex (mPFC) has been shown to play a central role in the regulation of social status in rodents (Wang et al., 2011; Zhou et al., 2018, 2017). The mPFC of rodents can be divided into dorsal (including the anterior cingulate cortex and the prelimbic cortex) and ventral (including the infralimbic cortex) regions (Dalley, Cardinal, & Robbins, 2004). In particular, the dorsal mPFC is thought to establish social status (Wang et al., 2011). Notably, activation of the dorsal PFC elevates the social rank of subordinates, and inactivation of the dorsal mPFC lowers the social rank of dominants in mice (Wang et al., 2011). AMPA (alpha‐amino‐3‐hydroxy‐5‐methyl‐4‐isoxazolepropionic acid) receptor‐mediated synaptic efficacy in dorsal mPFC pyramidal neurons is associated with these shifts, which indicates that social status is modulated by excitatory neurotransmission in the mPFC of mice (Park, Seo, Lee, Shin, & Kang, 2018; Wang et al., 2011). Another region that plays a key role in social hierarchy in rodents is the nucleus accumbens (NAc). Recent studies have revealed that mitochondrial function in the medium spiny neurons in the NAc with dopamine receptors is involved in the social status of mice (Larrieu et al., 2017), and drugs targeting these neurons can change the social ranks of rats (van der Kooij et al., 2018). Zhou et al. (2017) revealed that the mediodorsal thalamus (MDT) input to the dorsal mPFC also plays a key role in the winner effect in mice. The synaptic strength in the MDT‐dorsal mPFC pathway is associated with social status and has been reported to be enhanced after repeated winning in mice (Zhou et al., 2017). In addition, the authors revealed that optogenetic long‐term depression (LTD) in the MDT‐dorsal mPFC synapses negated the sustained winning effect, while optogenetic long‐term potentiation (LTP) in the MDT‐dorsal mPFC synapses caused long‐lasting social rank elevation. These studies have provided detailed insights into synaptic plasticity associated with the winning effect; earlier reports focused on hormonal changes that occur after repeated victories (Qu & Dreher, 2018; Zhou et al., 2018). For example, Timmer, Cordero, Sevelinges, and Sandi (2011) reported that the mRNA expression levels of oxytocin in the medial nucleus of the amygdala were correlated with the establishment of social status. Moreover, recent primate studies have shown that the amygdala is a pivotal brain region to underlie social status (Munuera et al., 2018; Wellman, Forcelli, Aguilar, & Malkova, 2016). In nonhuman primates, dopamine D2 receptor expression in the striatum has been shown to be associated with higher social status in monkeys (Morgan et al., 2002; Yamaguchi, Lee, Kato, Jas, & Goto, 2017). Pharmacological investigations have revealed that administration of a D2 antagonist decreases the social rank of dominants in monkeys (Yamaguchi et al., 2017), which indicates that the D2 receptor is important to maintain social status. Serotonin has been implicated in the formation and maintenance of social status in monkeys (Raleigh, McGuire, Brammer, Pollack, & Yuwiler, 1991; Raleigh, McGuire, Brammer, & Yuwiler, 1984). Raleigh et al. (1984) found bidirectional modulation of social status by the 5‐HT system in monkeys; thus, social status affects 5‐HT levels in blood, and its levels are related to acquisition of social status. Noonan et al. (2014) revealed that the size of the dorsal raphe nucleus is larger in dominant monkeys than in subordinate monkeys. The dorsal raphe nucleus is known to be the origin of 5‐HT projection neurons.

Recent neuroimaging studies in humans have indicated that the dorsolateral prefrontal cortex (DLPFC), ventrolateral prefrontal cortex (VLPFC), ventromedial prefrontal cortex (VMPFC), intraparietal sulcus (IPS), amygdala, hippocampus, and striatum are the principal brain regions related to social status (Qu et al., 2017; Watanabe & Yamamoto, 2015). The lateral prefrontal cortex (LPFC), including the DLPFC and VLPFC, is involved in attention in humans (Miller & Cohen, 2001). Recent studies have shown that the LPFC exhibits stronger activation when humans pay attention to superiors in a social context (vs. a human player) compared with a nonsocial context (vs. a computer player) (Chiao et al., 2009; Farrow et al., 2011; Marsh, Blair, Jones, Soliman, & Blair, 2009; Zink et al., 2008). Thus, the LPFC is associated with social status information (status cues) in the attentional system, and, therefore, the LPFC may code social status as a part of the social norm (Chiao, 2010; Watanabe & Yamamoto, 2015). Ligneul, Obeso, Ruff, and Dreher (2016) reported that the VMPFC reacts specifically to competitive victories, while the striatum is deactivated in response to social defeats. In that study, the authors found that social dominance status and the associated prediction errors are encoded in the rmPFC and that stimulation of the rmPFC using transcranial direct current stimulation (tDCS) enhanced the relative proportion of victories over defeats in learning social dominance, leading to changes in the social rank (Ligneul et al., 2016). Thus, the mPFC (including the VMPFC and rmPFC) and the striatum play pivotal roles in learning social status through competitions (Ligneul et al., 2016; Qu et al., 2017). The IPS in humans is involved in magnitude judgments, such as those in the number comparison task (Dehaene, Piazza, Pinel, & Cohen, 2003; Zink et al., 2008). Chiao et al. (2009) revealed that the IPS is recruited during social status comparison. The amygdala is involved in perception, learning, formation, and maintenance of social status (Kumaran, Melo, & Duzel, 2012; Watanabe & Yamamoto, 2015; Zink et al., 2008). Zink et al. (2008) found that the amygdala is activated in unstable hierarchy conditions in which the ranking of participants could change according to the results of the game. Amygdala activity has been found to be correlated with the individual motivation to reach the top rank, which indicates that the amygdala is modulated by motivational inputs (Watanabe & Yamamoto, 2015; Zink et al., 2008). Kumaran et al. (2012) also found that activation of the amygdala was correlated with the confidence level in social status.

## 4 SOCIAL STATUS AND MTD

### 4.1 MTD

The idea of MTD emerged in Japan in the 2000s (Kato, Hashimoto, et al., 2016). While MTD is not an official medical term, the concept has become widely known in Japan. Individuals with MTD exhibit situation‐dependent depressive symptoms and a strong avoidant tendency (Kato, Hashimoto, et al., 2016; Kato & Kanba, 2017). They complain about their mental sickness and try to stay away from work or school, but once they have escaped from these situations, they are capable of enjoying their lives without any mental burden (Kato, Hashimoto, et al., 2016). MTD is frequently diagnosed as atypical depression, dysthymia, or personality disorder, but in most cases, it does not meet the diagnostic criteria for these conditions (Kato & Kanba, 2017; Kato et al., 2011). Thus, individuals with MTD are diagnosed as showing an adjustment disorder. Indeed, individuals with MTD are primarily young adults who cannot adjust to the traditional hierarchical social order (Kato & Kanba, 2017). They are very susceptible to social defeat induced by hierarchy in the social environment and are likely to experience social defeat as trauma (Kato & Kanba, 2017). Hence, they easily fall into social withdrawal, and at worst, *hikikomori* (Kato Kanba, & Teo, 2016, 2019). Their personality traits are characterized by avoidance and narcissism (Kato, Hashimoto, et al., 2016; Tarumi, 2005). Kato, Hashimoto, et al. (2016)) reviewed the details of MTD and proposed a novel diagnostic approach for MTD.

### 4.2 Potential mechanisms of MTD

**Figure 1:**
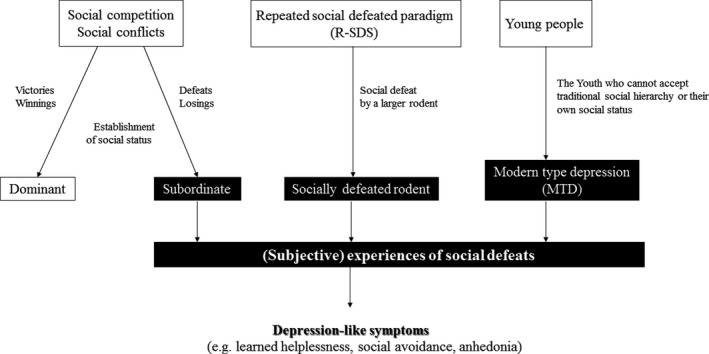
Subjective experiences of social defeat and depression‐like symptoms

When considering the pathology of MTD alongside the findings from studies of social status described above, we noticed that MTD could be attributed to social defeat when living in a society with a social hierarchy. In mice, CSDS causes depression‐like symptoms, such as social withdrawal, anhedonia, and immobility in the forced swim test (Venzala et al., 2012). CSDS is performed using two strains of mice with different sizes, thus forcing smaller mice to experience defeats in competitions (Golden et al., 2011). Social competitions generate winner/loser effects (Hsu et al., 2006), and accumulation of victories or losses establishes the social status (Drews, 1993; Hand, 1986). Therefore, considering the mechanisms associated with the establishment of social status, even among identical strains, living in a society with a social hierarchy may produce depression‐like symptoms and loss of motivation. As mentioned above, Horii et al. (2017) revealed that subordinate mice show depression‐like behaviors (e.g., learned helplessness) similar to those exhibited by mice in CSDS (Venzala et al., 2012). Assuming that losers may experience more trauma with defeats, much like patients with MTD (Kato & Kanba, 2017), they are likely to become subordinates through losing effects, at least subjectively, and to exhibit depression‐like symptoms such as social avoidance. MTD caused by an inability to accept the traditional hierarchical social order may also occur after social defeat experiences in life (Kato & Kanba, 2017), which suggests that MTD may be similar to the depression‐like behaviors of mice with CSDS or subordinate mice that have lost competitions (Figure 1). Therefore, the findings of animal experiments using CSDS or social status could be helpful to clarify the mechanisms underlying MTD by bridging basic neuroscience data to clinical phenotypes of it (Russo, Murrough, Han, Charney, & Nestler, 2012).

### 4.3 Social rank‐dependent susceptibility to psychosocial stress and its implications for MTD

**Figure 2:**
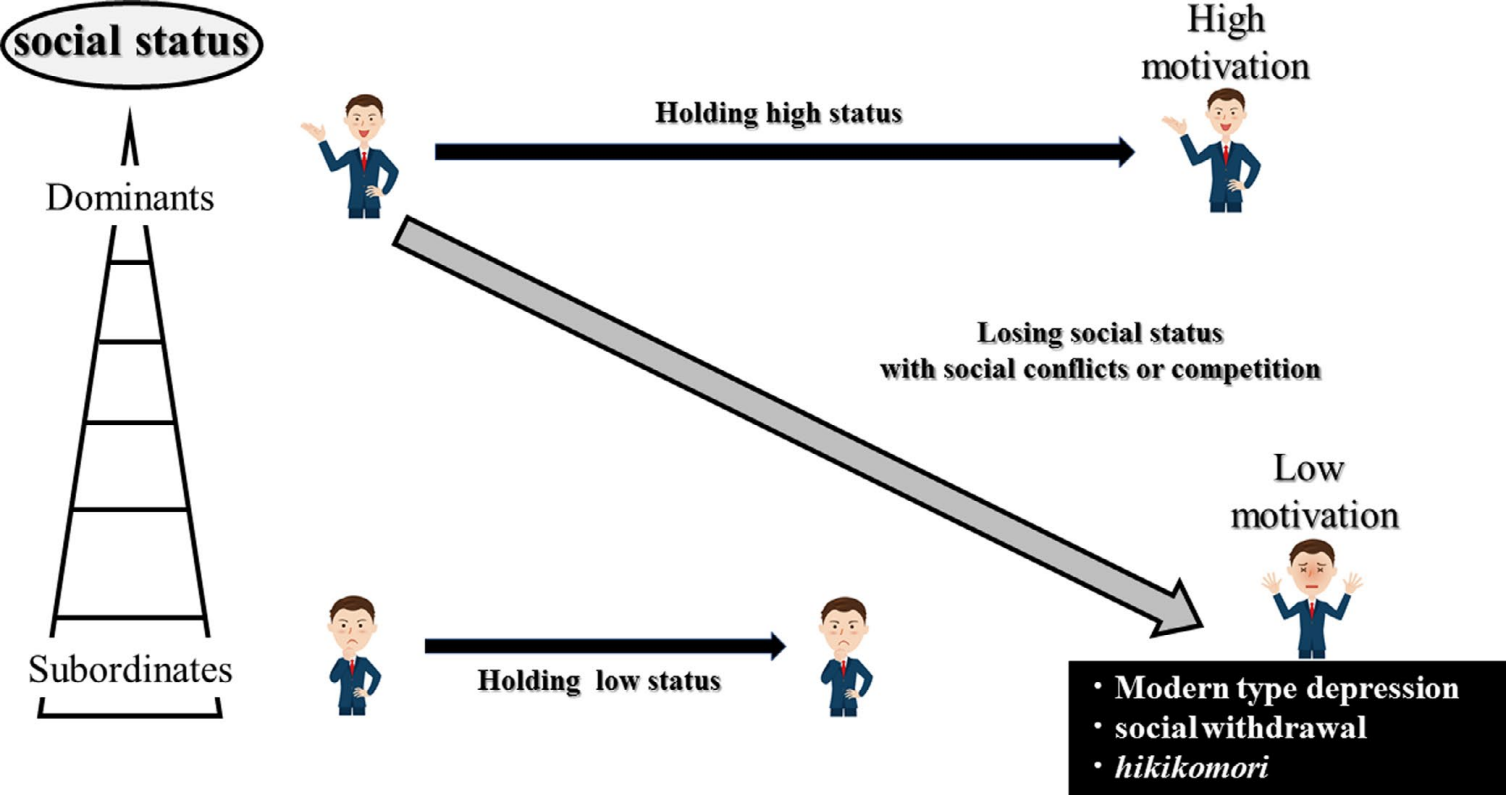
Changes of social rank and depression‐like symptoms

Given that individuals with MTD may exhibit susceptibility to social defeat (Kato & Kanba, 2017), investigating the susceptibility to CSDS in mice could help to uncover the neural mechanisms of MTD. Larrieu and colleagues sought to elucidate the relationships between social rank and susceptibility to CSDS in mice (Larrieu et al., 2017; Larrieu & Sandi, 2018). Unexpectedly, dominant mice were more susceptible to CSDS with social avoidance and depression‐like behaviors than subordinate mice (Larrieu et al., 2017). The authors proposed that this was because subordinate mice were used to social defeat during the establishment of social status, thus leading to more resilience to CSDS (Larrieu et al., 2017; Larrieu & Sandi, 2018). These findings could suggest that the effects of social defeat during the establishment of social status are similar to those of CSDS and that the subordinate mice may not feel such burdens of CSDS. Alternatively, the social status of captive mice may be despotic and maintained through frequent physical reassertion of dominance in which high‐ranking individuals tend to experience stress as described above (Sapolsky, 2005). Larrieu et al. also suggested that dominant mice were more susceptible to unpredicted defeats (Larrieu et al., 2017; Larrieu & Sandi, 2018). Interestingly, this phenomenon in mice may mimic the symptoms of MTD in humans (Figure 2). In modern human society, the youth tend to live with the *amae*, *kahogo*, and *yutori kyoiku* in Japan (Kato, Hashimoto, et al., 2016). *Amae* is a Japanese term for behaviors and emotions that can be defined as the “presumed acceptance of one's inappropriate behavior or request” (Yamaguchi & Ariizumi, 2006); *kahogo* and *yutori kyoiku* are Japanese terms that refer to the overprotectiveness of parents and a relaxed education with less content, respectively (Kato & Kanba, 2017; Sakurai, 2016). Unpredicted defeats may be their first experience of being defeated for the young after becoming members of the society. The youth, who have relished the *amae*, *kahogo*, and *yutori kyoiku*, could keep a high rank, at least subjectively, without competition with other individuals or social defeats (Kato, Hashimoto, et al., 2016; Kato & Kanba, 2017). In addition to these adolescent environments and their education system, the narcissistic traits underlying MTD (Kato, Hashimoto, et al., 2016; Tarumi, 2005) may contribute to the susceptibility to social defeat, since younger adults with narcissistic traits may believe that they are superior than others, regardless the objective facts (Caligor, Levy, & Yeomans, 2015). These findings imply that the composition of modern human society resulting in MTD may be similar to that of societies in which repeated and physical reassertion is required to hold social status or social status is unstable in some primates (Sapolsky, 2005).

### 4.4 Resilience to social defeat stress and potential interventions to MTD

Animal experiments with invasive approaches, such as optogenetic stimulation, have been performed to determine the specific neural circuits involved in social defeat stress (Zhou et al., 2017). Although such approaches are useful to identify the neural circuits and neurochemical mechanisms of vulnerability to CSDS in animals, it is difficult to apply these methods to humans. As of now, only antianxiety agents have been shown to increase resilience to CSDS in rats (van der Kooij et al., 2018). Resilience is defined as the individual ability to recover from or adjust to difficulties in life (Davydov, Stewart, Ritchie, & Chaudieu, 2010). It is desirable that people voluntarily act to increase their own resilience to social stress. One recent study has found that voluntary exercise increases resilience to CSDS in mice (Mul et al., 2018), which indicates that voluntary exercise may be an effective and useful intervention to increase resilience to social defeat stress in humans. Gut microbiota, diet, and the immune system have also been implicated in resilience to CSDS in mice (Ambree, Ruland, Scheu, Arolt, & Alferink, 2018; Aubry et al., 2018; Kingston et al., 2018; McKim et al., 2016; Nie et al., 2018; Szyszkowicz, Wong, Anisman, Merali, & Audet, 2017). Improvement in eating habits may help change the immune system through alterations of microbiota, which may in turn increase resilience to social defeat stress in humans, especially given the strong correlations between the immune system and resilience to CSDS (Ambree et al., 2018; Aubry et al., 2018; McKim et al., 2016; Nie et al., 2018; Szyszkowicz et al., 2017). tDCS is a noninvasive intervention that has already been applied for the treatment of depression in clinical practice (Fregni et al., 2006; Nitsche, Boggio, Fregni, & Pascual‐Leone, 2009). Ligneul et al. (2016) reported that tDCS of the rmPFC shifts the values of victory and defeat for dominance learning in humans, which suggests that tDCS may be a useful tool to improve the mental disorders induced by social hierarchal order. Psychotherapy is generally also thought to be useful to overcome avoidance. Kato and Kanba (2017) developed an effective rehabilitation program for MTD called “Re‐Work,” in which participants learn skills to adjust to Japanese working places in which social hierarchal order is emphasized.

## 5 CONCLUSION

In Japan, MTD has recently become a critical topic for adolescent mental health (Kato & Kanba, 2017). Individuals who cannot accept their own social status on the basis of the Japanese traditional hierarchal order can exhibit depression‐like behaviors, such as learned helplessness and social withdrawal, which is considered to indicate the presence of MTD (Kato, Hashimoto, et al., 2016; Kato & Kanba, 2017). Given that MTD is a subtype of adjustment disorders that are resistant to psychotropic drugs (O'Donnell, Metcalf, Watson, Phelps, & Varker, 2018), medication is likely to be ineffective. In this article, we have reviewed the potential mechanisms of MTD from the standpoint of social status using knowledge gained mainly from captive animals and humans and have proposed that a shared pathobiology mechanism underlies social status and MTD. For a better understanding of the dynamics of MTD, further studies are warranted to validate the biological mechanisms of social status in feral nonhuman primates with no obvious social hierarchy in addition to those of captive animals. These findings suggest that physical interventions such as voluntary exercise, diet, tDCS, psychotherapy, and the absence of psychotropic drugs could hold strong potential as therapeutic interventions for MTD.

## CONFLICT OF INTEREST

There are no conflicts of interest.

## Data Availability

Data sharing is not applicable to this article as no new data were created or analyzed in this study.
